# Mechano-regulated surface for manipulating liquid droplets

**DOI:** 10.1038/ncomms14831

**Published:** 2017-04-04

**Authors:** Xin Tang, Pingan Zhu, Ye Tian, Xuechang Zhou, Tiantian Kong, Liqiu Wang

**Affiliations:** 1 Department of Mechanical Engineering, the University of Hong Kong, Pokfulam, Hong Kong SAR, China; 2 HKU-Zhejiang Institute of Research and Innovation (HKU-ZIRI), Hangzhou, Zhejiang 311300, China; 3 College of Chemistry and Environmental Engineering, Shenzhen University, 3688 Nanhai Avenue, Shenzhen 518060, China; 4 Guangdong Key Laboratory for Biomedical Measurements and Ultrasound Imaging, Department of Biomedical Engineering, Shenzhen University, 3688 Nanhai Avenue, Shenzhen 518060, China

## Abstract

The effective transfer of tiny liquid droplets is vital for a number of processes such as chemical and biological microassays. Inspired by the tarsi of meniscus-climbing insects, which can climb menisci by deforming the water/air interface, we developed a mechano-regulated surface consisting of a background mesh and a movable microfibre array with contrastive wettability. The adhesion of this mechano-regulated surface to liquid droplets can be reversibly switched through mechanical reconfiguration of the microfibre array. The adhesive force can be tuned by varying the number and surface chemistry of the microfibres. The *in situ* adhesion of the mechano-regulated surface can be used to manoeuvre micro-/nanolitre liquid droplets in a nearly loss-free manner. The mechano-regulated surface can be scaled up to handle multiple droplets in parallel. Our approach offers a miniaturized mechano-device with switchable adhesion for handling micro-/nanolitre droplets, either in air or in a fluid that is immiscible with the droplets.

## Abstract (web summary)

Droplet manipulation is an essential task for designing microfluidic platforms such as lab-on-chip devices. Here Tang *et al*. develop a non-wettable mesh with reversible liquid adhesion controlled by mechanically inserting wettable pillars which allows for effective and rapid droplet manoeuvring.

The effective manipulation of tiny liquid droplets, surrounded by either a carrier liquid^1^,^2^ or air^3^,^4^,^5^, is critical in diverse fields such as microfluidics, microassays and medical diagnosis^6^,^7^,^8^,^9^,^10^,^11^. Techniques have been developed for manoeuvring the droplets on a planar surface. Various types of external forces, including electric, magnetic and acoustic, have been applied for droplet actuation^12^,^13^,^14^,^15^,^16^. The actuated droplets usually wet their substrates during transport, leading to loss of liquid and substrate contamination^6^. Another technique, termed the liquid marble method, facilitates liquid transfer without the wetting of the substrate through the coating of aqueous liquid droplets with hydrophobic powders^3^. However, such powder encapsulation of droplets complicates their manipulation and impedes the visualization and detection of the fluid inside^15^. These techniques work successfully for droplets on a flat surface in air but encounter difficulties in attempts to manipulate the droplets either on an arbitrary surface or in an immiscible liquid.

Biological surfaces in nature interact with droplets in diverse manners. For instance, the bumpy backs of desert beetles can harvest water drops from foggy wind^17^, and the nipple arrays on the compound eyes of mosquitoes can prevent the accumulation of water drops^18^. Inspired by these fascinating organisms, artificial surfaces have been fabricated to be highly adhesive for capturing droplets and non-adhesive for releasing them. Superhydrophobic surfaces with specific liquid adhesion properties have been developed based on responsive materials, for example^19^,^20^,^21^. Their interfacial chemistry and roughness change in response to external stimuli such as pH, light and temperature to alter the mobility of droplets^19^,^20^,^21^. However, such a stimulus-responsive strategy is *ex situ* in nature and it can take minutes to hours to switch the adhesion of such a surface^22^. For example, the external temperature must rise from 23 to 75 °C to cause a droplet to switch from a rolling to a pinned state on a substrate coated with PDMS-4OCB, a side-chain liquid crystal polymer^20^. The stimuli involved may also denature some bioactivities and thus be inapplicable for the handling of droplets containing bioactive materials^23^,^24^. Hence, it is highly desirable to develop superhydrophobic surfaces whose adhesion to liquid droplets can be switched in real time and without relying on stringent stimuli.

Meniscus-climbing insects such as *mesovelia* have evolved to possess retractable hydrophilic ungues on the tips of their hydrophobic-hair-covered tarsi^25^. These semiaquatic insects can walk on water because of their non-wetting tarsi. To depart for land, they distort the water's surface by using their hydrophilic claws and thereby gain the lateral capillary force necessary to enable them to ascend the meniscus slope at the water's edge^25^.

The tarsi of meniscus-climbing insects inspired our work on the development of a mechano-regulated surface (MRS) for the manoeuvring of liquid droplets. The proposed MRS is created by distributing movable quartz microfibres with a strong liquid affinity over a non-wettable background polyester mesh coated with graphene nanoplatelets. Unlike conventional liquid-repellent surfaces, the solid/liquid adhesion on our MRS can be switched *in situ* by reconfiguring the microfibres on the non-wetting mesh. Micro-/nanolitre liquid droplets on the MRS can be reversibly switched between pinned and roll-off states within 1 s. The solid/liquid adhesion can be readily and precisely tuned by manipulating the formation of capillary bridges through modification of the wettability and the number of microfibres. The proposed MRS can be used for the preparation and deposition of nanolitre liquid droplets. Our MRS strategy, which enables liquid handling in air or in an immiscible fluid, shows a high potential for application in many fields, such as biotechnology and microfluidics.

## Results

### Design of the mechano-regulated surface

**Figure 1:**
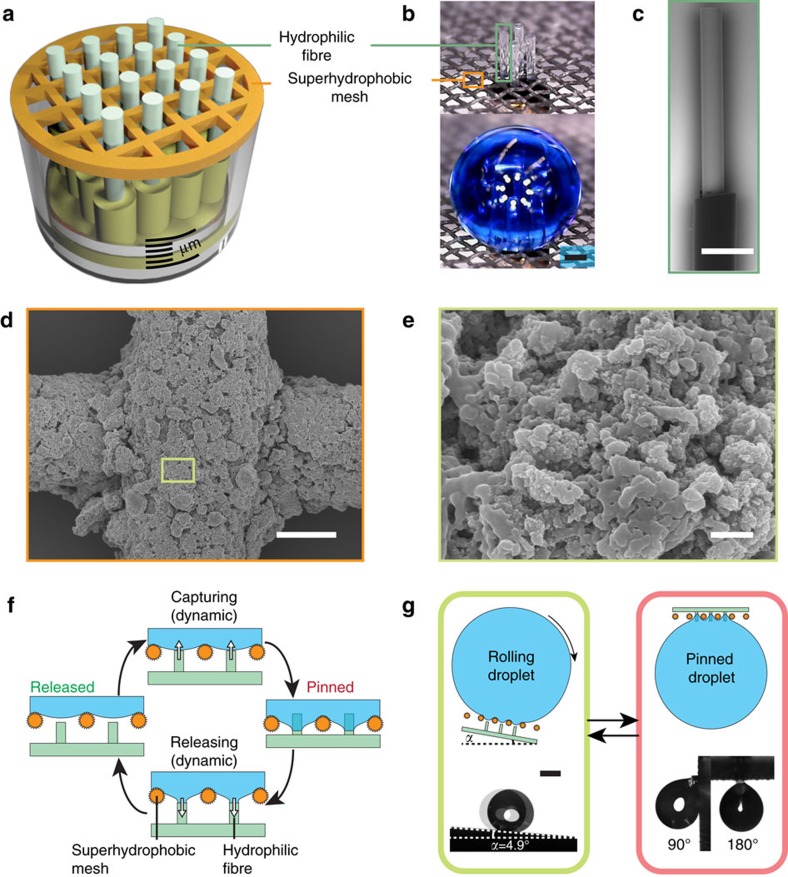
The design and principle of the MRS. (**a**) Schematic illustration of the assembly of an MRS, which consists of a superhydrophobic mesh and an array of hydrophilic microfibres. (**b**) Optical micrographs showing the structure of a four-fibre MRS; a water droplet (stained blue) is locked onto the MRS. Scale bar, 300 μm. Scanning electron microscopy images of (**c**) a hydrophilic microfibre wrapped in a protective jacket, scale bar, 300 μm; (**d**) a superhydrophobic mesh coated with graphene nanoplatelets, scale bar, 30 μm; and (**e**) a typical magnified region in (**d**), scale bar, 500 nm. (**f**) A side-view schematic illustration of the mechanism of the MRS: the hydrophilic array (green) advances towards the superhydrophobic mesh (orange) (Capturing) until the array protrudes from the pores of the mesh, at which time the water droplet is pinned (Pinned); when the array is retracted through the mesh (Releasing), the surface returns to its non-wettable state, allowing the water droplet to roll (Released). (**g**) Schematic illustrations and optical micrographs showing the rolling and pinned states, respectively, of a water droplet on an MRS. Scale bar, 1 mm.

To create a superhydrophobic surface with movable domains of high surface energy, we designed microfibres with a high aspect ratio that can mechanically protrude and retract through a superhydrophobic mesh (Fig. 1a–c). A piece of polyester mesh was used as the background surface (see Supplementary Note 1 and Supplementary Fig. 1 for details). To prevent wicking of the liquid droplets (Supplementary Note 2 and Supplementary Fig. 2), we covered the mesh with a superhydrophobic coating consisting of graphene nanoplatelets and polydimethylsiloxane. After curing the coating, the mesh was composed of threads with a diameter of approximately 65 μm; the pore size of the mesh was determined by the inter-thread distance, 200 μm. Therefore, the distance between the centres of neighbouring pores was 265 μm. The surface of the mesh became rough because of the micro- and nanoscale hydrophobic structures (Fig. 1d,e), endowing the treated mesh with a water contact angle of 151°. A small bundle of peeled optical fibres was used as the movable microfibres. With a diameter of 125 μm, the tips of the fibres were smooth with exposed fused quartz that was inherently hydrophilic. Protective jackets were used to maintain the spacing between the centres of neighbouring fibres at 255 μm (approximately the same as the distance between the centres of neighbouring mesh pores, 265 μm) such that each fibre could protrude out of a pore in the mesh. To study the influence of the wettability on the adhesion properties of the surface, we also prepared an MRS using hydrophobic microfibres. The hydrophilic quartz fibres were silanized to make them hydrophobic through the application of octadecyltrichlorosilane (OTS). The superhydrophobic mesh was attached to the top of a syringe while the hydrophilic/hydrophobic fibre array was fixed to the plunger of the syringe using commercial adhesives. As the plunger advanced or retreated, the array of quartz fibres either protruded into or retracted from the superhydrophobic mesh. The step size of the advancing plunger was controlled by a *z*-axis translational stage with a precision on the scale of micrometres.

The on/off switching of the surface's adhesion to water droplets is controlled by moving the array of microfibres. The superhydrophobic mesh allows a water droplet to roll easily. As the array of regulating fibres protrudes from the mesh, local wettable domains emerge and attract water droplets (Fig. 1f, capturing). Once seized by such a domain, the water droplet remains pinned on the surface even when the mesh is tilted to 90° or 180° (Fig. 1g). This pinning is maintained unless an external force such as gravity exceeds the capillary force, thus leading to cohesion failure of the droplet. The superb stability of the droplet adhesion permits any arbitrary manoeuvring of the attached droplet. By retracting the fibre array, the mesh is restored to its superhydrophobic state (Fig. 1f, releasing). Thus, the previously adhesive surface becomes a non-sticky one (Fig. 1f, released), allowing the droplet to be released to a targeted location in a loss-free manner. Therefore, the adhesion of a water droplet on the MRS switches instantaneously when the mechanical movement of the plunger is completed.

### Characterization of the adhesive performance of MRSs

**Figure 2:**
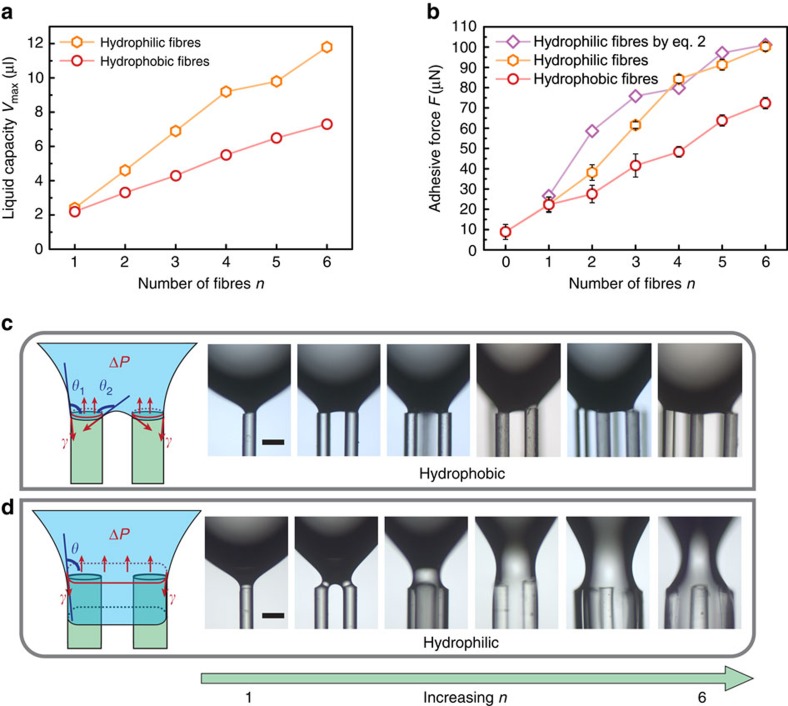
The adhesive performance of the MRS. (**a**) The liquid capacity of an MRS increases as the number of regulating fibres increases. (**b**) The measured adhesive force of an MRS increases as the number of fibres increases. The red and orange lines represent the measured adhesion of MRSs with hydrophobic and hydrophilic fibres, respectively, based on ref. 26. The purple line represents the estimated adhesion of an MRS with hydrophilic fibres based on equation (2). The error bars indicate the standard deviations over five independent measurements. A series of optical microscopy images showing (**c**) the capillary bridges with receding contact lines on MRSs with numbers of hydrophobic fibres varying from 1 to 6 and (**d**) the thinning capillary bridges between the droplets and MRSs with numbers of hydrophilic fibres varying from 1 to 6. Scale bars, 200 μm.

The capillary adhesive force of the MRS can be tuned by varying the number and surface chemistry of the regulating microfibres. As the number of microfibres increases, the maximum volume of a water droplet (liquid capacity) that the MRS can hold vertically against gravity also increases, as shown in Fig. 2a. The liquid capacity of a hydrophilic-fibre MRS is higher than that of a hydrophobic-fibre MRS. For example, a 6-fibre MRS with hydrophilic fibres has a maximum liquid capacity of 11.8 μl, higher than the 7.3 μl capacity of an MRS with hydrophobic fibres (Fig. 2a). When the liquid volume exceeds the liquid capacity, the droplet will rupture and fall because the gravitational force will overcome the capillary force.

To quantitatively estimate the capillary adhesive strength of the MRS for liquid droplets, we pulled the captured water droplet away from the MRS until its detachment from the MRS. The adhesive force was then determined by considering the vertical force balance of the lower part of the pulled droplet immediately before its detachment (Supplementary Note 3 and Supplementary Fig. 3)^26^. The adhesive force of the plain superhydrophobic mesh was found to be approximately 8.9 μN, indicating the ready detachment of water droplets. Then, we measured the adhesion of MRSs with different numbers of fibres of both the hydrophobic and hydrophilic types (Fig. 2b). Consistent with the liquid capacity data, the measured adhesion increased with an increasing number of fibres. For example, the adhesion of the MRS increased drastically from 8.9 μN to 72.3 μN or 100.2 μN as the number of hydrophobic or hydrophilic fibres, respectively, was increased from 0 to 6.

The strength of the adhesive force is determined by the formation of capillary bridges between the droplets and the MRS (Fig. 2c,d). The capillary bridges that form on a hydrophobic-fibre MRS differ from those on a hydrophilic-fibre MRS. When a droplet is pulled away and is about to detach from a hydrophobic-fibre MRS, the water contact line begins to recede with a receding contact angle *θ*_r_ on the fibre facet (Fig. 2c) in accordance with the Gibbs criterion^27^. The receding angle *θ*_r_ was measured to be 96.4° for our hydrophobic fibres. The contact angle varies in the range of 96.4°⩽*θ*<180° on the periphery of the quartz fibres because of the interaction between neighbouring capillary bridges. Thus, the maximal capillarity-based adhesion, which would require *θ*=90°, cannot be reached. The capillary adhesion of a hydrophobic-fibre MRS at the base of the capillary bridges can be calculated as follows^28^,^29^:


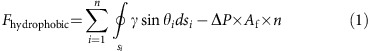


where *n* is the total number of quartz fibres; *s*_*i*_, *A*_f_ and *θ*_*i*_ are the circumference, cross-sectional area and localized contact angle of the *i*th fibre, respectively; and *γ* and Δ*P* are the surface tension of the water and the Laplace pressure, respectively. For a fibre number of less than or equal to six, the contact angles on the fibres remain similar. As the number of fibres (*n*) increases, the number of capillary bridges increases, leading to a higher capillary adhesive force.

For a hydrophilic-fibre MRS, water will infiltrate the microfibre bundle to create a liquid column. As the droplet is stretched away, a capillary bridge with a thinning fluid neck is formed (Fig. 2d). Before the pinch-off of the capillary bridge, the angle (*θ*) between the water surface and the fibre facet can reach 90°, yielding the maximum capillarity-based adhesive force, as follows:


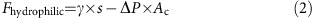


where *s* and *A*_c_ are the cross-sectional circumference and area, respectively, of the liquid column at the base of a capillary bridge.

The cross-sectional circumference (*s*) and area (*A*_c_) can be obtained by assuming that the base of the capillary bridge is a rounded-corner polygon with corners defined by fibres (see Supplementary Note 4 and Supplementary Fig. 4 for details). We can then use equation (2) to estimate the adhesion force and compare the strengths of its two components: the surface tension and the Laplace pressure. As the number of fibres increases from 1 to 6, the ratio of the Laplace pressure over the surface tension varies from 0.07 to 0.11, 0.176, 0.224, 0.248 and 0.278, respectively. The effect of the Laplace pressure becomes stronger as the number of fibres increases and can be neglected only when the fibre number is low (fewer than three). The estimated adhesive forces agree well with the data measured using the method presented in ref. 26. A higher number of fibres in the outermost region results in a thicker capillary bridge, giving rise to a higher capillary adhesive force. If the number of fibres is not too large (fewer than or equal to six, as in the present work), all fibres can be arranged to lie at the periphery of the droplet and thus to contribute to the formation of the capillary bridge. The adhesive force is therefore proportional to the number of fibres. For a sufficiently large fibre number *n*, however, some fibres will unavoidably be located in the interior of the bridge, such that the adhesive force will level off as the number of fibres increases. We limited our measurements of the adhesive force to within a fibre number of 6, which is sufficiently large for capturing tiny droplets whose radii are much smaller than the corresponding capillary length^30^.

### Manipulation of aqueous liquid droplets using an MRS in air

**Figure 3:**
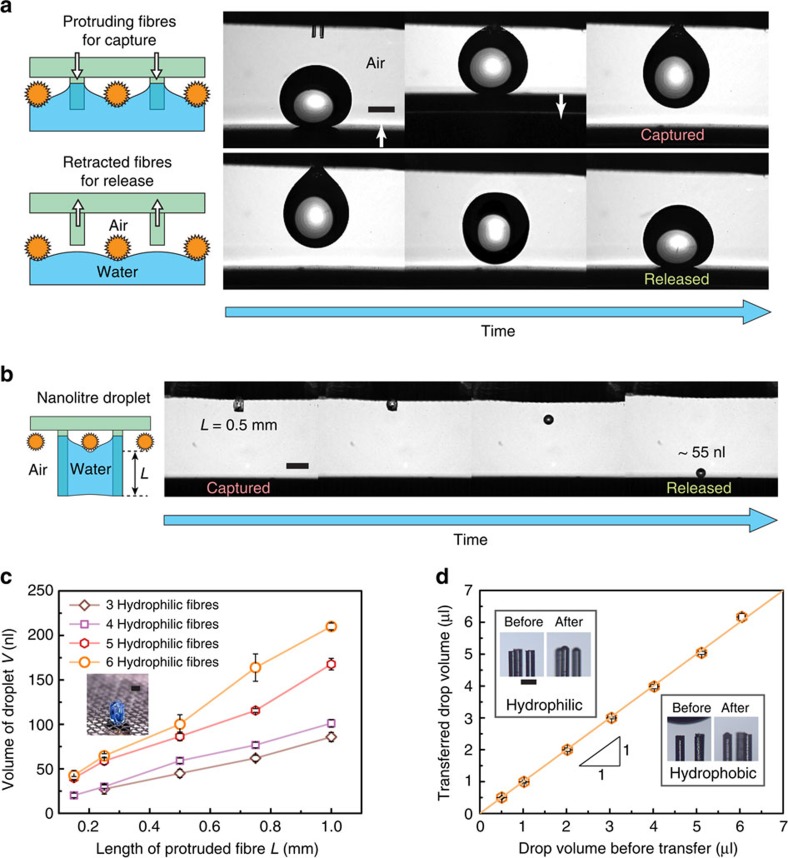
The manipulation of aqueous droplets in air using an MRS. Schematic illustrations and time-sequence images showing the capture and complete release of water droplets of (**a**) 10 μl and (**b**) 55 nl onto a superhydrophobic substrate in air. Scale bar, 1 mm. (**c**) The volume of the water droplet prepared using a hydrophilic-fibre MRS increases as the protrusion length of the fibres increases. The inset presents an optical micrograph showing a stained water column trapped by four hydrophilic fibres. Scale bar, 300 μm. The error bars indicate the standard deviations over five independent measurements. (**d**) The drop volume remains the same before and after transfer using a 4-hydrophilic-fibre MRS. The insets present optical microscopy images showing almost no water residue on the regulating fibres after the transfer process. Scale bar, 200 μm. The error bars indicate the standard deviations over five independent measurements.

The *in situ* switchable adhesion of an MRS enables the real-time manipulation of tiny droplets, including capture, transport and release. We demonstrated the capture and release of a microlitre water droplet in air, as shown in Fig. 3a. A water droplet of 10 μl was picked up using the adhesive MRS. To disengage the droplet, the protruding fibres were withdrawn from the mesh. Consequently, the droplet readily detached from the MRS and was thereby dispensed onto a superhydrophobic substrate, which is otherwise difficult to deposit onto because of its non-sticky property of superhydrophobicity (Supplementary Movie 1, Supplementary Note 5 and Supplementary Fig. 5). With a typical fibre protrusion length of 500 μm and a motion speed of 500–600 μm s^−1^, the adhesion switching of the MRS was completed within 1 s.

In addition to handling water droplets of microlitre size, the hydrophilic-fibre MRS can also be used to aliquot nanolitre droplets from a bulk solution and then to distribute these tiny droplets, a task beyond the capability of typical micropipettes. Here, we demonstrated the successful preparation and release of 55 nl water droplets using the hydrophilic-fibre MRS (Fig. 3b). We used the hydrophilic-fibre MRS to extract water from a bulk solution to prepare nanolitre droplets. The extracted water wetted the hydrophilic fibres protruding from the mesh and thus became trapped between the fibres, as shown schematically in Fig. 3b and in the inset photograph in Fig. 3c. Subsequently, the fibres were withdrawn, upon which the superhydrophobic mesh repelled the extracted water, releasing a nanolitre water droplet. The volume of such a prepared water droplet corresponds to the space between the protruding fibres, which is determined by the number and protrusion length of the fibres. By using a *z*-axis translational stage to precisely control the protrusion length, we showed that water volumes from 20 nl to 200 nl could be extracted and then dispensed by 3-fibre to 6-fibre MRSs, respectively (Fig. 3c). The minimum volume of 20 nl is constrained by the size of the mesh pores. Therefore, a smaller water droplet could be prepared if thinner fibres and a mesh with smaller pores were to be used. We did not use hydrophobic-fibre MRSs to extract water from bulk solution since the water would not become trapped between hydrophobic fibres.

More importantly, the transport of such tiny droplets is nearly loss-free, which is particularly advantageous for handling precious liquid samples. To evaluate the liquid loss during the MRS transfer process^31^, we compared the volumes of droplets before and after transfer using a 4-hydrophilic-fibre MRS (Fig. 3d). To simplify the estimation of the droplet volume, a silicone-oil-impregnated silicon wafer was used as the deposition substrate, which ensured circular three-phase contact lines of the deposited droplets^32^,^33^,^34^. Droplets with volumes varying from 0.5 to 6 μl were transported using the MRS. No noticeable change in the droplet volume was found, as shown in Fig. 3d. To further confirm that the liquid loss is nearly zero during the transfer process, we observed the withdrawal of the fibre array under a microscope (Supplementary Note 6 and Supplementary Fig. 6). For the hydrophilic-fibre MRS, we observed that a minute amount of water remained on the top facet of each microfibre (Fig. 3d, insets). This water residue, measured to be roughly 0.15 nl, is due to the pinch-off of thin water bridges^35^. By contrast, for an MRS with three hydrophobic fibres, we observed a perceptible water residue with a volume of roughly 0.07 nl on only one fibre facet. The highly reduced liquid retention of hydrophobic fibres (0.023 nl per fibre) is ascribed to the easy receding of the three-phase contact line on the hydrophobic fibre surface. In contrast to the liquid retention of manual pipettes, which is on the scale of 10 nl or even 100 nl, the liquid loss with an MRS is negligible. This negligible loss could be further reduced by using thinner fibres.

To demonstrate the application of this nearly loss-free process, we used an MRS to assist in low-concentration detection (Supplementary Note 7 and Supplementary Fig. 7)^36^,^37^. Initially, we used our MRS to capture a 9-μl droplet of a highly diluted (66.7 nM) Rhodamine 6G (R6G) solution, which is not observable under a fluorescence microscope. The pendant droplet was allowed to evaporate until the volume shrank to 40 nl; the concentrated nanolitre droplet was subsequently deposited on a glass slide, and the fluorescence could be readily detected.

### Manipulation of oil droplets using an MRS underwater

**Figure 4:**
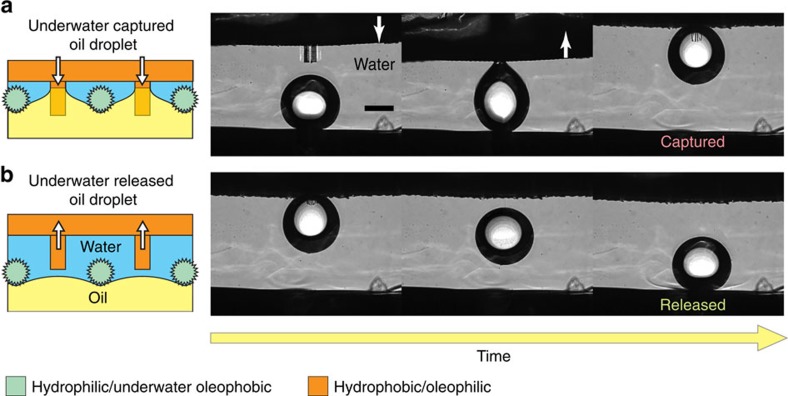
The manipulation of oil droplets underwater using an MRS. Schematic illustrations and time-sequence images showing (**a**) the underwater capture of a 5-μl droplet of DCE using an MRS with hydrophobic fibres and (**b**) the subsequent release of the DCE droplet underwater. Scale bar, 1 mm.

If its surface chemistry is modified, our MRS is also capable of manipulating oil droplets underwater. We designed an oil-manipulating MRS consisting of an underwater superoleophobic mesh and regulating fibres that are preferentially wetted by oil. A layer of water-retaining gel, calcium alginate, was coated onto the mesh. Since the absorbed water in the hydrogel acts as an intermediate phase that repels oil, the mesh is rendered superoleophobic underwater^38^. Hydrophobic fibres silanized with OTS were used as the regulating fibres. These fibres have a high affinity towards oil and thus provide underwater adhesion to oil droplets. We demonstrated that a droplet of 5 μl of 1,2-dichloroethane (DCE) could be successfully captured and released using the modified MRS, as shown in Fig. 4a,b. The DCE droplet was first placed on an underwater superoleophobic substrate, a piece of silicon wafer coated with calcium alginate. As the superoleophobic MRS with oleophilic fibres approached the DCE droplet, the droplet was attracted and firmly clung to the MRS. Then, the oil droplet was lifted up from the substrate as the MRS was drawn away. When the fibres were withdrawn from the superoleophobic mesh, the DCE droplet was in contact only with the superoleophobic mesh and thus instantly became detached and fell back onto the substrate (Fig. 4b and Supplementary Movie 2). Therefore, our MRS can be used for the selective manipulation of oil droplets in an immiscible aqueous liquid, a task that is otherwise difficult to achieve.

### MRS durability

**Figure 5:**
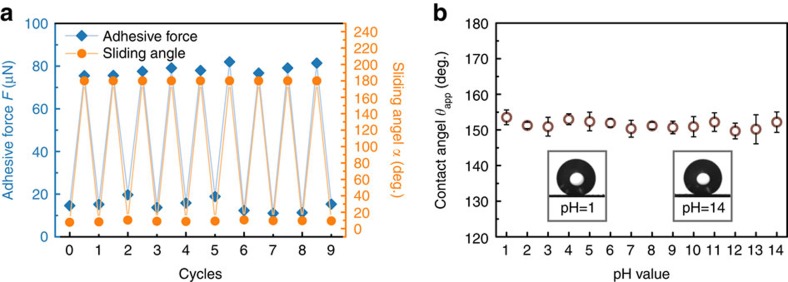
The durability of an MRS. (**a**) A nine-cycle measurement of reversible switching between the adhesive and slippery states for a 4-hydrophilic-fibre MRS. For the sliding angle measurements, 5-μl water droplets were used. (**b**) The contact angle of a water droplet on an MRS remains constant for pH values varying from 1 to 14, demonstrating the excellent chemical resistance of the MRS. The error bars indicate the standard deviations over five independent measurements. The insets present the silhouettes of droplets with pH values of 1 and 14 on the superhydrophobic mesh.

Our MRS can be used repeatedly for the capture and release of liquid droplets. To show this, we tested its cycling durability. The reversible switching of the MRS between adhesive and slippery states was demonstrated for over nine cycles, as shown in Fig. 5a. For each cycle, the sliding angles for a 5-μl water droplet in the adhesive and slippery states were 180° (pinned) and less than 10°, respectively. The durability and cyclic reversibility of the MRS enable repeated usage and multistep operations for droplet-based applications.

To demonstrate the compatibility of the MRS with various environments, we also tested the chemical resistance of the superhydrophobic coating over a wide range of pH values varying from 1 to 14. Over this wide range of pH values, aqueous droplets on an MRS without fibres exhibited a stable non-wetting Cassie state with contact angles larger than 150° (Fig. 5b). The chemical resistance of the MRS is due to the chemical stability of its coating, a mixture of graphene nanoplatelets and polydimethylsiloxane^39^,^40^.

### Application and scalability of MRSs for droplet manipulation

**Figure 6:**
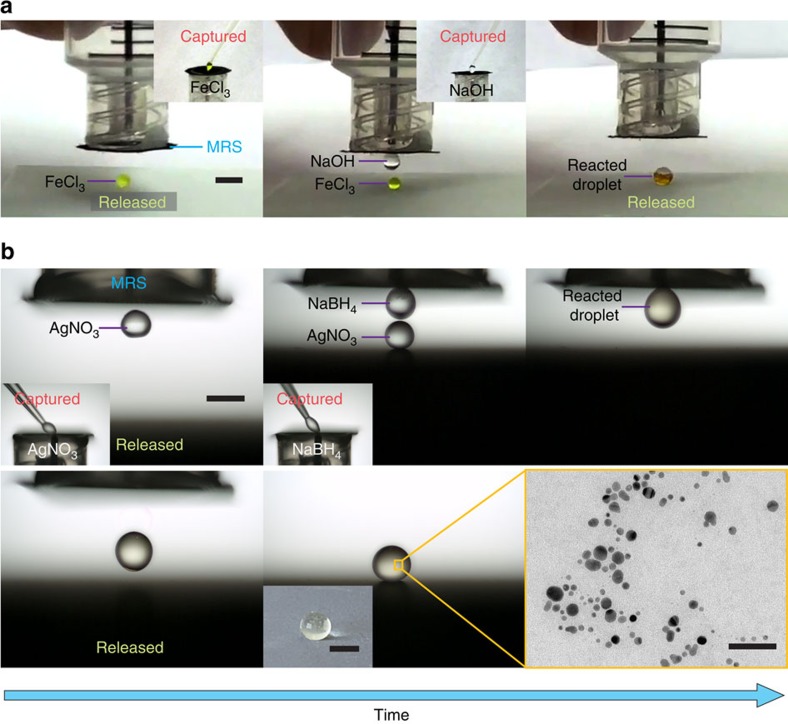
MRS-assisted micro-reactors. (**a**) Time-sequence images showing an MRS-based process using droplet-based micro-reactors for the generation of Fe(OH)_3_ precipitates. Scale bar, 3 mm. (**b**) Time-sequence images showing an MRS-based process using droplet-based micro-reactors for the synthesis of silver nanoparticles. Scale bars, 2 mm. Also presented in a transmission electron microscopy image showing the synthesized silver nanoparticles. Scale bar, 50 nm.

The effective manipulation of tiny droplets that is possible using our MRS is highly useful for their application as micro-reactors. As models for demonstration (Fig. 6a and Supplementary Movie 3), we triggered a chemical reaction between FeCl_3_ and NaOH droplets serving as micro-reactors using our MRS. We captured a 2-μl droplet of an FeCl_3_ (0.75 M) solution and released it onto a superhydrophobic pedestal. Subsequently, a 4-μl droplet of an NaOH (1.5 M) solution was captured and forced to mix with the deposited droplet of FeCl_3_ solution. The reaction was initiated, as indicated by the formation of brown Fe(OH)_3_ precipitates on the pedestal. In addition to the generation of precipitates, MRS-assisted micro-reactors can also be used for the effective synthesis of nanoparticles. As a demonstration, we synthesized silver nanoparticles by mixing AgNO_3_ and NaBH_4_ droplets (Fig. 6b)^41^. We captured a 2-μl droplet of an AgNO_3_ (0.5 mM) solution and released it onto a superhydrophobic pedestal. Subsequently, an ice-cold droplet consisting of 2 μl of an NaBH_4_ (0.015 M) solution was captured and forced to coalesce with the deposited droplet of AgNO_3_ solution. The mixing of the two droplets was enhanced by applying a gentle nitrogen flow (10 cm s^−1^) surrounding the pendant droplet (Supplementary Movie 4). Silver nanoparticles were successfully synthesized, as shown by the light yellow colour of the merged droplet on the pedestal (Fig. 6b). When stabilized with a polyvinylpyrrolidone aqueous solution (0.03%, w/w), the silver nanoparticles were found to have an average diameter of 7.87 nm, as observed via transmission electron microscopy (Fig. 6b; see Supplementary Note 8 and Supplementary Fig. 8). The lossless transport of tiny droplets using our MRS enables precise control of the reactant volume and, thus, the micro-reaction precision that is widely demanded in diagnostics, analytical chemistry and biotechnology.

**Figure 7:**
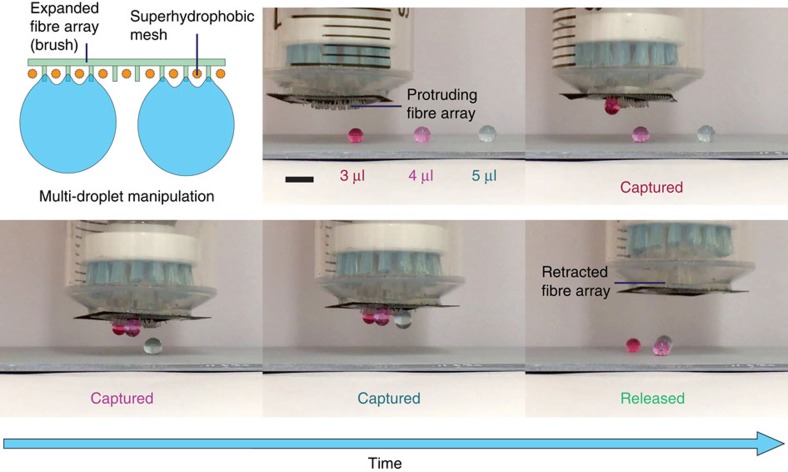
Manipulation of multiple droplets using a scaled-up MRS. Schematic illustration and time-sequence images showing the in-parallel manipulation of multiple droplets using an MRS with a larger-scale fibre array. The scaled-up MRS successively captures three aqueous droplets (dyed red, magenta and blue) of different volumes and then releases them simultaneously. Scale bar, 3 mm.

Our MRS can also be scaled up to manipulate multiple droplets simultaneously, which is vital for applications requiring a high throughput. To demonstrate this, we used a brush made of Dupont Tynex bristles as a larger-scale array of flexible fibres. This scaled-up MRS was used to manoeuvre three individual water droplets in parallel (Fig. 7). As the MRS with the protruding brush bristles approached the water droplets, it successively captured the 3-, 4- and 5-μl water droplets. To release these pinned droplets, we retracted the brush through the superhydrophobic mesh to dispense them onto the non-wettable substrate below (Supplementary Movie 5). This demonstrated scalability makes our MRS promising for high-throughput and large-scale droplet handling. Furthermore, the fibre array of an MRS can be divided into subsections that can be individually controlled to enable independent droplet-state switching for droplets located in different regions.

## Discussion

Inspired by the tarsi of meniscus-climbing insects, we have developed an MRS consisting of a stationary liquid-repelling mesh and movable liquid-wettable microfibres. Its adhesion to liquid can be rapidly, reversibly and effectively switched in an *in situ* manner. It can be used to capture and release both water and oil droplets with volumes down to the nanolitre scale precisely and effectively, which is a must in many applications, such as chemical/biological microassays, multi-compartment droplet preparation and diagnosis. The effortless and reliable manoeuvring of liquid droplets enabled by the proposed MRS is effective for assisting in the detection of highly diluted material and in operations using droplet-based micro-reactors. The scalable liquid-handling capability of the MRS may potentially be applied in fields where efficiency is highly valued, such as drug discovery.

## Methods

### Fabrication of mechano-regulated surfaces

The polyester mesh was purchased from Wanwei Mesh (Taizhou, China). Single-mode optical fibres with a diameter of 125 μm were purchased from Yangtze Optical Fibre and Cable (Hubei, China). All materials were used as received. The following chemicals were used as received without further purification: graphene nanoplatelets (Cheap Tubes Inc., Grade 3), SYLGARD 184 silicone elastomer kit (Dow Corning), ethanol (Merck, ≥99.8%), diethyl ether (Fisher Chemical, anhydrous, ≥99%), acetone (Merck, ≥99.8%), OTS (Sigma-Aldrich, ≥90%), toluene (Sigma-Aldrich, anhydrous, 99.8%), poly(ethylenimine) (Aladdin, M.W. 10000, 99%), sodium alginate (Sigma-Aldrich), calcium chloride (Sigma-Aldrich, anhydrous, ≥97%), silicone oil (Sigma-Aldrich, 5 cSt), silver nitrate (Sigma-Aldrich, ≥99%), sodium borohydride (99%), polyvinylpyrrolidone (Aladdin, M.W. 10,000), methylene blue (EKEAR, ≥98%), Direct Red 80 (Aladdin, AR), sulforhodamine B (Sigma-Aldrich, 75%) and Rhodamine 6G (Sigma-Aldrich, ∼95%). The polyester mesh was first rinsed with deionized water, acetone and ethanol and then dried with nitrogen. The dried mesh was subsequently dipped and coated in a coating solution. The coating solution was prepared by mixing polydimethylsiloxane (0.5 g, with 10% crosslinker) and graphene nanoplatelets (0.5 g) with diethyl ether (8 ml) and ethanol (7 ml) at room temperature. The coating was then cured at 80 °C for 2 h to complete the process.

The superoleophobic polyester mesh was fabricated as described elsewhere^38^. The mesh was first dip coated in a poly(ethylenimine) solution (10%, w/v). After drying, the mesh was then dip coated in a sodium alginate solution (2%, w/v). Finally, the mesh was immersed in a calcium chloride solution (1 M) for 10 min.

Optical fibres cut using a fibre cleaver were used as the regulating fibres. The protective jackets were peeled away from the fibre tips using a fibre stripper. The exposed quartz tips of the optical fibres were cleaned with ethanol and treated with an ethanol flame. To make the fibres hydrophobic, a silanization process was applied by immersing the quartz tips in an OTS-in-toluene solution (10 mM) for 15 min. Then, the quartz fibres were subsequently flushed with toluene and cured in a furnace at 100 °C for 30 min.

The fabricated mesh was fixed on the top of a plastic syringe (TERUMO, 5 ml) using commercial adhesives. The fibres were bundled in a metal tube and adhered to the front of the syringe plunger. The plunger was then inserted into the barrel of the syringe. By pushing and pulling the plunger, the quartz fibres could be made to either penetrate or retract through the mesh. For the underwater manipulation of oil droplets, a hole was cut in the syringe to prevent the generation of negative pressure during the withdrawal of the plunger. For the manipulation of multiple droplets, a brush made of Dupont Tynex bristles was adhered to the front of the syringe plunger. Similarly, by pushing and pulling the plunger, the flexible bristles could be made to either penetrate or retract through the pores of the mesh.

### Instruments and characterization

The contact angles were measured using a self-built measuring system and were analysed using the DropSnake plugin in ImageJ (1.46r). The evaporation method was used to measure the receding contact angles^42^. First, a water droplet (8 μl) was deposited on the surface; then, after evaporation for 20 min at an ambient temperature of 25 °C and a relative humidity of 70%, the receding contact angle was measured using our self-built system. To measure the receding contact angles on OTS-modified quartz fibres, quartz slides (Chutian Inc., JGS2) with a material composition similar to that of the quartz fibres were modified and used. The sliding angles were measured using a tilting stage (SIGMAKOKI, GOH-40A35). The droplet volume used to measure the sliding angles was ∼5 μl (unless otherwise specified).

The assembly of the MRSs was observed using an optical microscope (GAOSUO). For visualization, the water droplets on the MRSs were dyed with methylene blue. The nanostructures of the coated mesh surface were characterized using a field-emission scanning electron microscope (Hitachi, S-4800). To measure the maximum liquid capacity, we measured the maximum water droplet volume that could be captured and held by the MRSs. The droplet volume was controlled using a quantitative micropipette (Dragon Lab, 0.5–10 μl). The adhesive forces of the MRSs were evaluated by deforming a water droplet of 3 μl in volume. The capillary bridges and water residue on the fibre bundles were observed using an inverted optical microscope (Nikon, Eclipse TS100).

To evaluate the liquid loss during transfer, the shapes of droplets before and after transport were analysed using ImageJ, and the volumes of the droplets were then calculated using Pappus's centroid theorem. A silicone-oil-impregnated surface was used to ensure that the three-phase contact lines of the deposited droplets were circular^32^. The surface was fabricated as follows: silicone oil (10 μl) was deposited on a silicon wafer, followed by baking on a hot plate (Polish, P-10) at 300 °C for 180 s. The residual oil was removed by rinsing with acetone. Finally, a thin layer of silicone oil was deposited by spin coating silicone oil at 3,000 revolutions per minute for 30 s using a spin coater (IMECAS, KW-4B).

The process of manipulating and mixing micro-/nanolitre droplets for micro-reactor applications was recorded using a high-speed camera (Phantom, Miro 110) coupled with a camera lens (Sigma, 30 mm/F1.4/DC/HSM). The fibre bundle was fixed on a manual *z*-axis translation stage (Yaxin, LZ60) with a precision of 10 μm to control the protrusion length of the fibres. The volumes of the nanolitre-scale water droplets were also calculated using Pappus's centroid theorem. The synthesized silver nanoparticles were characterized using a transmission electron microscope (Philips, CM 100). The UV-vis absorption spectrum of the silver nanoparticle colloid was measured using a UV-vis spectrophotometer (Thermo Scientific, NanoDrop 2000). For the manipulation of multiple droplets, water droplets of 3, 4 and 5 μl in volume were dyed red, pink and blue with Direct Red 80, sulforhodamine B and methylene blue, respectively, for visualization.

### Data availability

The authors declare that the data supporting the findings of this study are provided in the article and its Supplementary Information.

## Additional information

**How to cite this article:** Tang, X. *et al*. Mechano-regulated surface for manipulating liquid droplets. *Nat. Commun.* **8,** 14831 doi: 10.1038/ncomms14831 (2017).

**Publisher's note**: Springer Nature remains neutral with regard to jurisdictional claims in published maps and institutional affiliations.

## Supplementary Material

Supplementary Information Supplementary Figures, Supplementary Notes and Supplementary References

Supplementary Movie 1 The capture and release of a water droplet in air.

Supplementary Movie 2 The capture and release of an oil droplet (1, 2-dichloroethane) underwater.

Supplementary Movie 3 The manipulation of droplets as micro-reactors.

Supplementary Movie 4 The enhanced mixing process by introducing a gentle nitrogen flow around the droplet.

Supplementary Movie 5 The parallel manipulation of multiple water droplets of different volumes.
