# Supplementary material for: Mechano-regulated surface for manipulating liquid droplets
Source: Nat Commun. 2017 Apr 4;8:14831. doi: 10.1038/ncomms14831 (PMC5382277; doi:10.1038/ncomms14831)
Supplement: Supplementary Information — Supplementary Figures, Supplementary Notes and Supplementary References [file ncomms14831-s1.pdf]

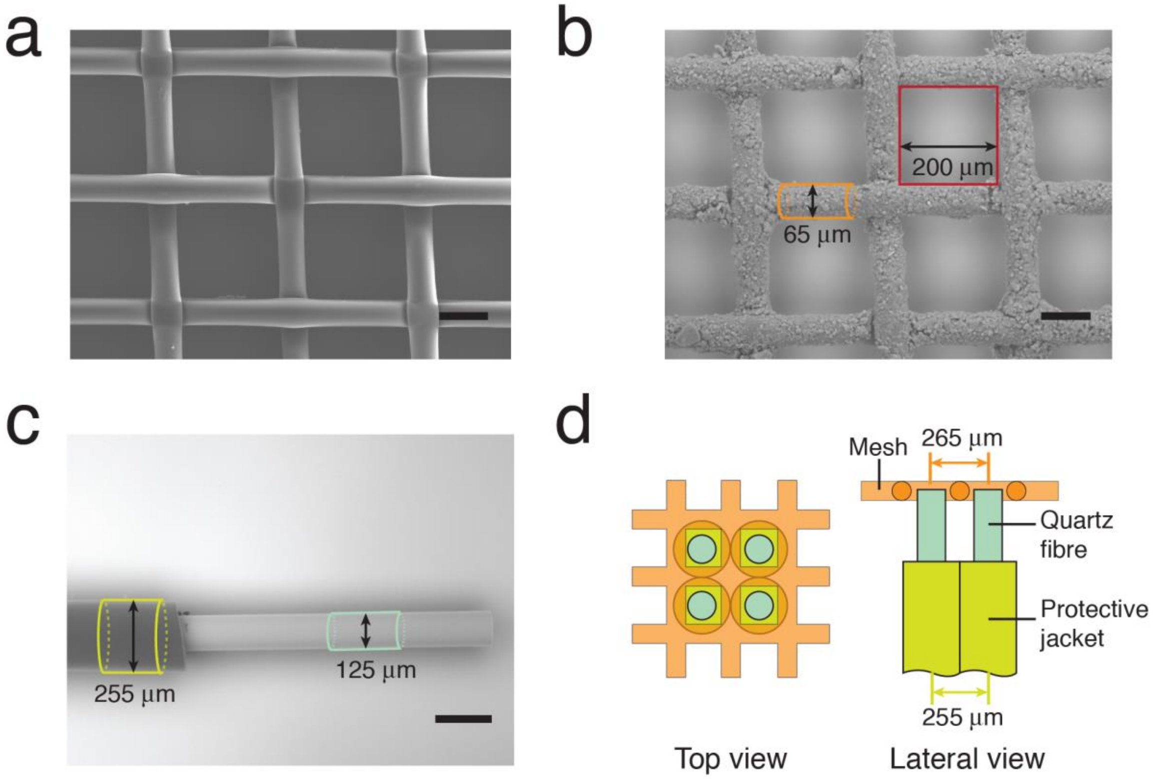

Supplementary Figure 1 | The fabrication of mechano-regulated surfaces. **(a)** Scanning electron microscopic (SEM) image of a polyester mesh before coating. Scale bar, 100 μm. **(b)** SEM image showing that the typical pore size and the thread diameter of the mesh after superhydrophobic coating are 200 μm and 65 μm, respectively. Scale bar, 100 μm. **(c)** SEM image showing that the diameter of the regulating optical microfibre and its protective jacket are 125 μm and 255 μm, respectively. Scale bar, 200 μm. **(d)** Schematics showing the assembly of the mechano-regulated surfaces (MRS).

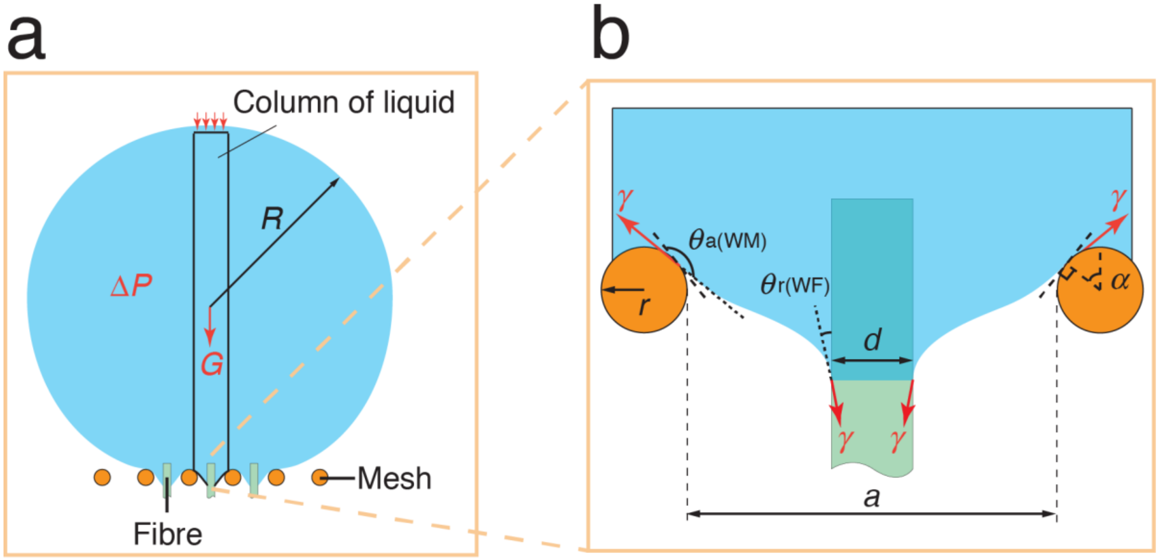

Supplementary Figure 2 | The static pinning state of a water droplet on the MRS. Schematic of **(a)** a water droplet pinned on a MRS, **(b)** the water contact line on the fibre and the background superhydrophobic mesh.

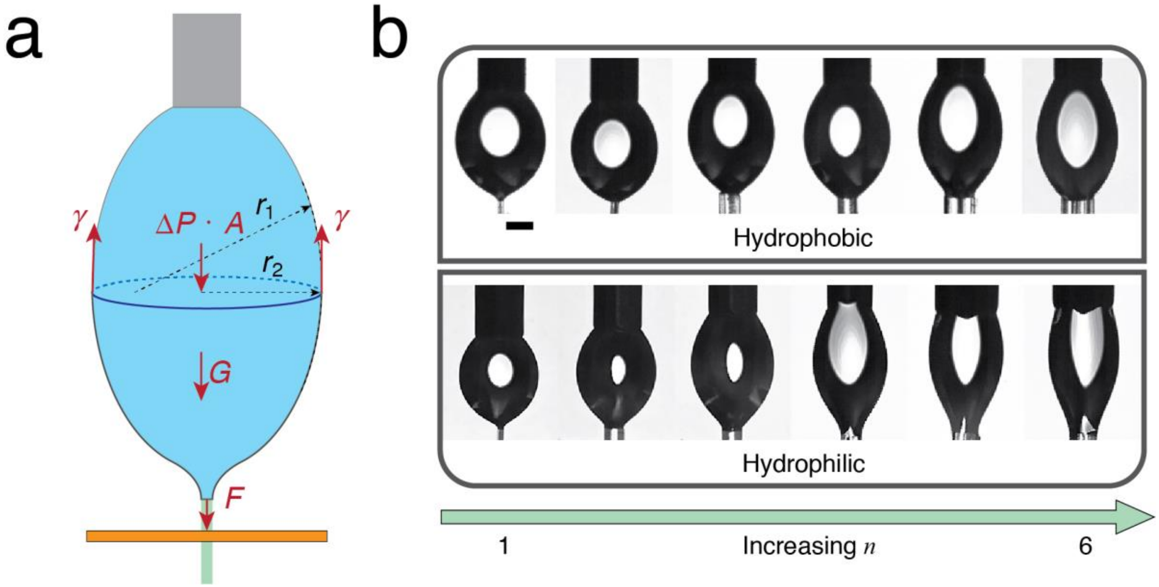

Supplementary Figure 3 | The method for measuring adhesive forces of the MRS. **(a)** Schematic showing the force balance of the lower part of a stretched water droplet. The adhesive force F is calculated by a force balance with surface tension force, Laplace pressure force, and gravitational force. **(b)** Snapshots of pulled water droplets on the MRS right before detachment. Scale bar, 0.5 mm.

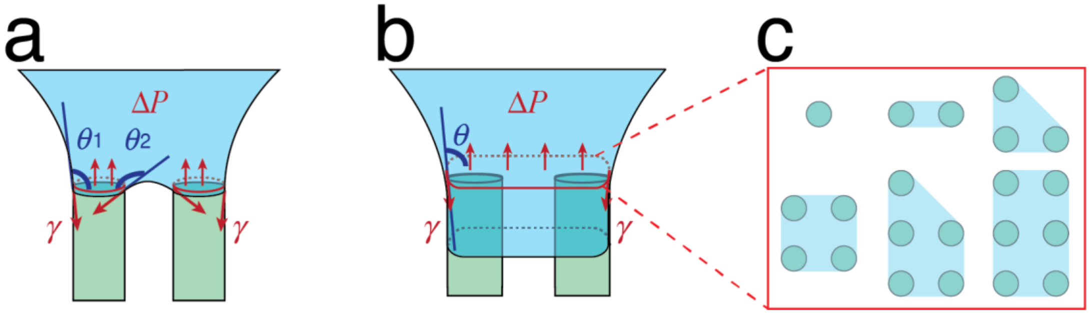

Supplementary Figure 4 | Shapes of the capillary bridges. Schematics showing the forces acting upon the base of the capillary bridge on **(a)** hydrophobic fibres and **(b)** hydrophilic fibres, respectively. **(c)** The schematic showing the cross sections of the capillary bridges, A_c, for the hydrophilic-fibre MRS with the fibre number varying from 1 to 6.

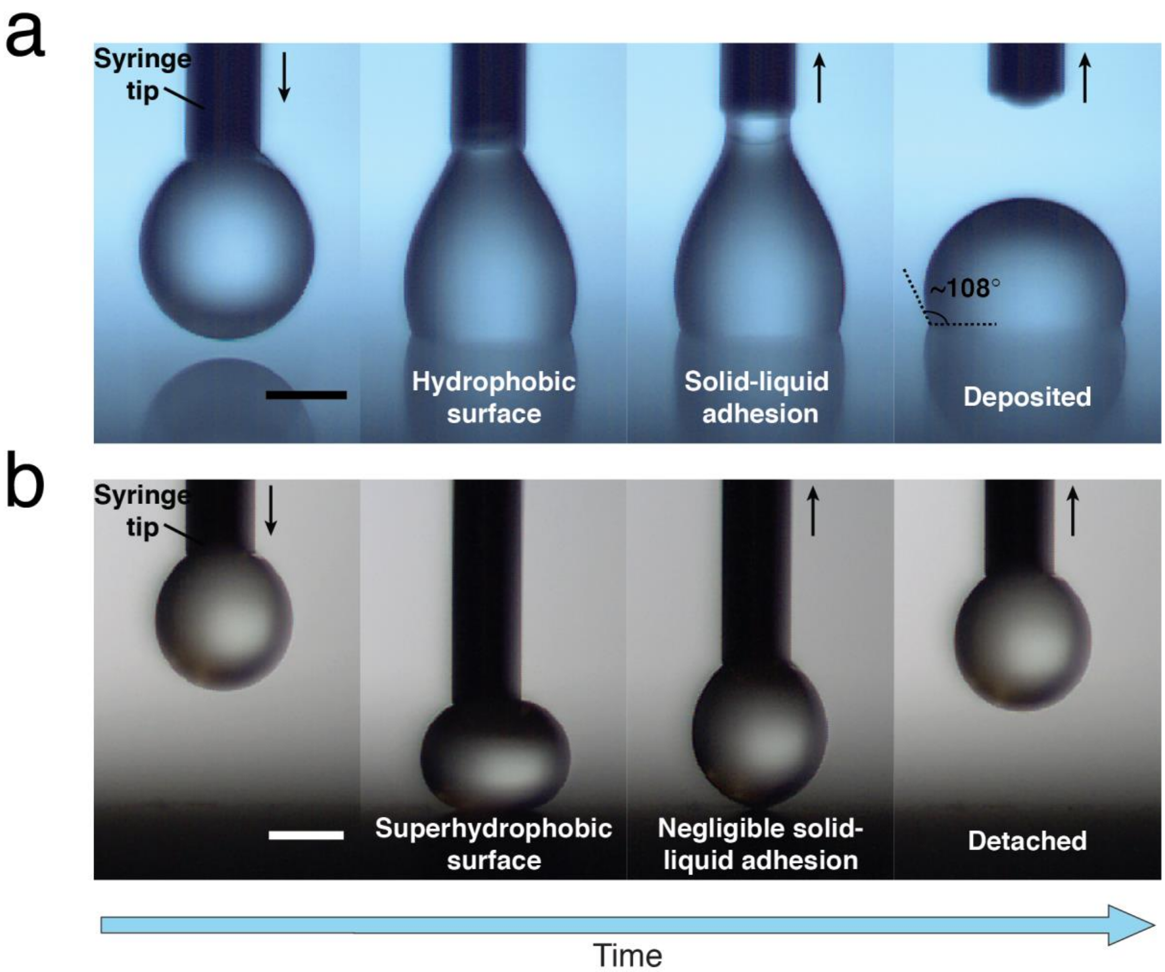

Supplementary Figure 5 | Deposition of water droplets. Time-sequence images showing **(a)** the deposition of a water droplet on a hydrophobic surface, and **(b)** the detachment of a water droplet from a superhydrophobic surface due to the negligible solid-liquid adhesion. Scale bars, 1 mm.

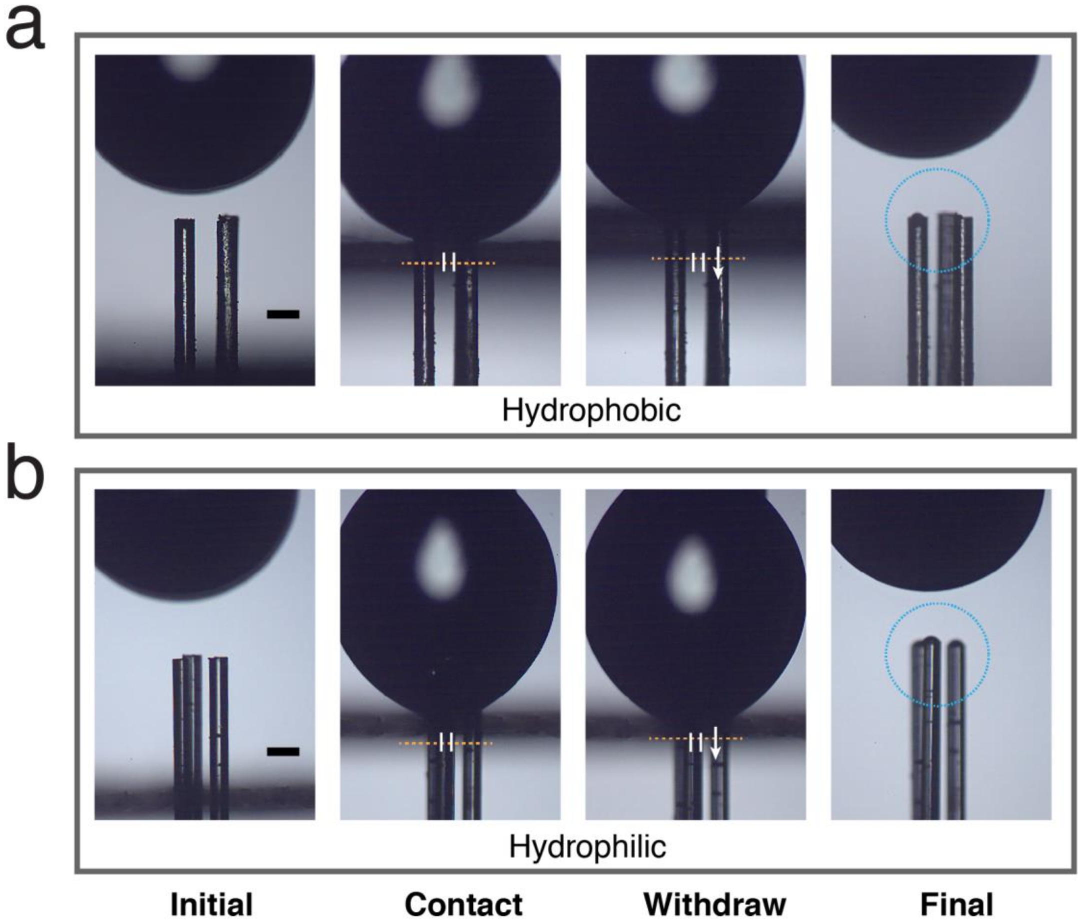

Supplementary Figure 6 | Withdrawal of fibres in MRS. Series of optical micrographs showing the fibre withdrawal process for the MRS with **(a)** hydrophobic fibres, and **(b)** hydrophilic fibres, respectively. Scale bars, 200 μm.

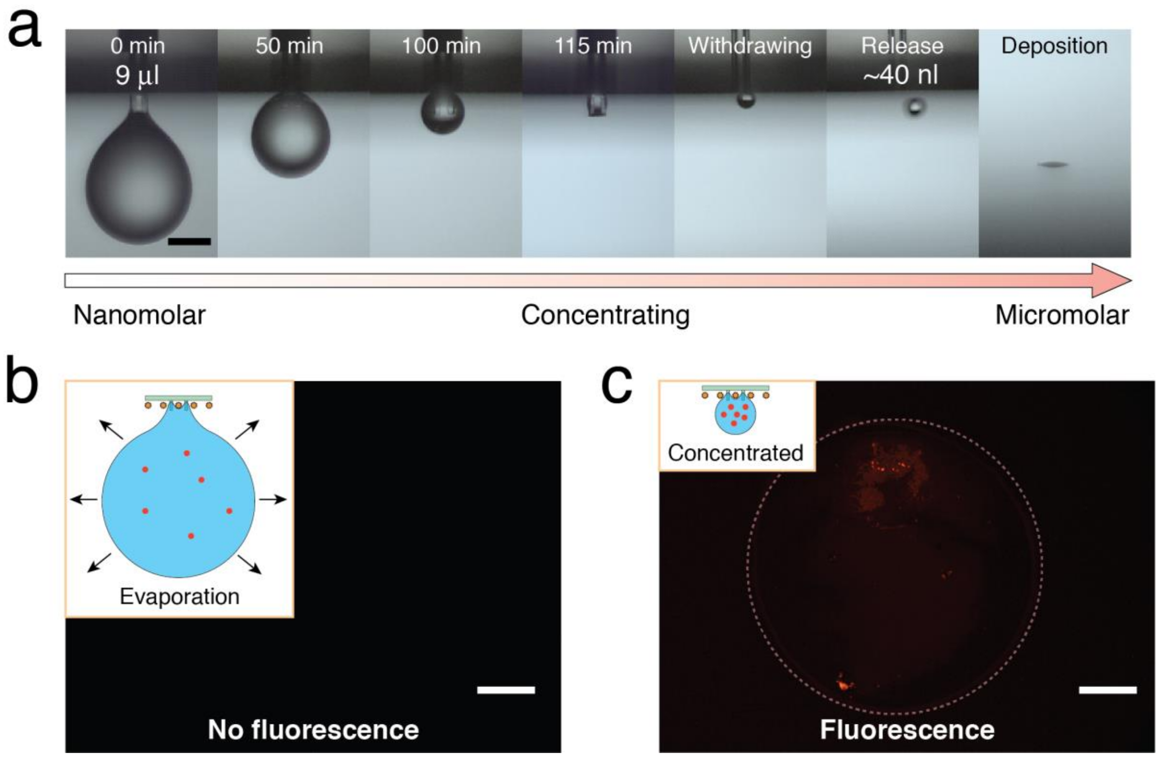

Supplementary Figure 7 | Assisting detection of highly-diluted chemicals with MRS. (a) Time-sequence images showing the evaporation and deposition process of rhodamine 6G (R6G) water droplet. Scale bar, 1 mm. **(b)** Fluorescence microscope image of a dried 9 μl water droplet dyed by 66.7 nM R6G; no fluorescence was observed due to the low concentration of R6G. Scale bar, 200 μm. **(c)** By water evaporation, the R6G concentration increased and the fluorescence was observed, indicating a successful detection. Scale bar, 200 μm.

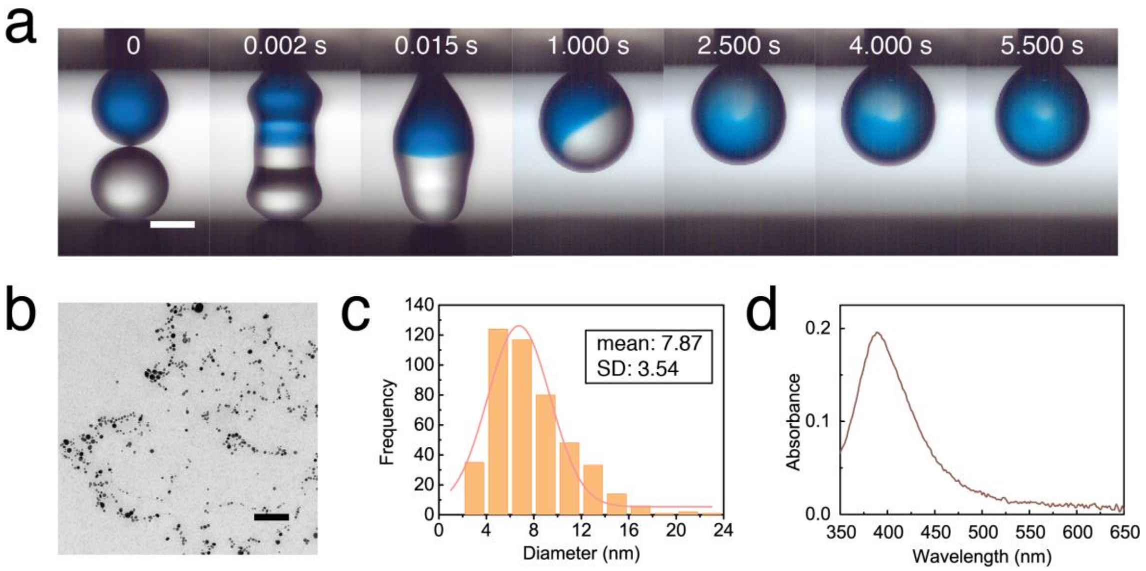

Supplementary Figure 8 | Mixing process for droplet-based micro-reactor. (a) Time-sequence images showing the mixing process of a coalesced droplet. Scale bar, 1 mm. **(b)** TEM image of the synthesized silver nanoparticles. Scale bar, 100 nm. **(c)** TEM-derived size distribution of the silver nanoparticles; the red curve is the Gaussian fitting curve. **(d)** UV-vis absorption spectrum of the silver nanoparticle colloidal suspension with a light yellow colour.

Supplementary Note 1-The assembly of mechano-regulated surfaces

To fabricate the MRS, a polyester mesh coated with superhydrophobic graphene nanoplatelets-based mixture and peeled optical microfibres were used. As illustrated in Supplementary Fig. 1, after coating, the mesh had a thread diameter of roughly 65 μm, and a square mesh pore length of about 200 μm. The quartz optical fibre had a diameter of 125 μm with its lower part wrapped in protective jackets. By tightly bounding the protective jackets of the fibres, the inter-fibre distance was about the same as the inter-pore distance of the mesh. Therefore, the matched size facilitates the protrusion and retraction of the fibre array.

Supplementary Note 2-The superhydrophobic background mesh

To pin the water droplet firmly on the MRS, the mesh should be sufficiently hydrophobic to prevent the wetting and wicking of the water droplet through the mesh. We can deduce the hydrophobic requirement of the mesh by considering a column of liquid inside the droplet as illustrated in Supplementary Fig. 2a. The force balance among the surface tension, Laplace pressure and gravitational force is as following:

$$\begin{aligned}\gamma\sin(\alpha+\theta_{\mathrm{a(WM)}}-\pi)\times[8r(1-\sin\alpha)+4a]=\gamma\cos\theta_{\mathrm{r(WF)}}\times\pi d+\frac{2\gamma}{R}\times[2r(1-\sin\alpha)+a]^2 \\ +\rho g[2r(1-\sin\alpha)+a]^2\times 2R\end{aligned} \quad (1)$$

where γ is the surface tension of water; α is the angle indicating the position of the contact line on the mesh threads; $\theta_{\mathrm{a(WM)}}$ is the advancing water contact angle on the mesh surface; *r* is the radius of the mesh threads; *a* is the inter-thread distance; $\theta_{\mathrm{r(WF)}}$ is the receding water contact angle on the quartz fibre surface; *d* is the diameter of the quartz fibres; ρ is the density of water; *g* is the gravitational acceleration; *R* is the radius of the water droplet.

Taking $\alpha=90°$, the Supplementary Equation (1) can be simplified as following:

$$-\gamma\cos\theta_{\mathrm{a(WM)}}\times 4a=\gamma\cos\theta_{\mathrm{r(WF)}}\times\pi d+\frac{2\gamma}{R}\times a^2+\rho g a^2\times 2R \quad (2)$$

Thus, using the parameters in our case ($d=125\ \mu\mathrm{m}$, $a=200\ \mu\mathrm{m}$, $R=1\ \mathrm{mm}$, $\theta_{\mathrm{r(WF)}}\approx 30°$), $\theta_{\mathrm{a(WM)}}$ is calculated to be about 122.59°.

Supplementary Note 3-The measurement of adhesive forces

The adhesive forces of MRS were measured by deforming water droplets and analysing their deformed shapes as describe in ref. 1[1]. In details, as shown in Supplementary Fig. 3, a pendant water droplet with volume of 3 μl on micro-syringe tip was brought to contact with MRS and then pulled away. By increasing the pulling distance, the droplet gradually deformed. Right before the droplet detached from the MRS, the outline of the deformed droplet was recorded and analysed to obtain the adhesive force.

Considering the force balance of lower part of water droplets, we have:

$$F_\gamma - \Delta P \times A - G - F = 0 \qquad (3)$$

where F_γ is the surface tension force calculated based on $F_\gamma = 2\pi r_2 \gamma$, ΔP is the Laplace pressure inside the droplet which is calculated based on $\Delta P = \gamma(\frac{1}{r_1} + \frac{1}{r_2})$, A is the cross-sectional area of the droplet, G is the gravitational force of lower part of the droplet, F is the adhesive force between the droplet and MRS. The adhesive force was calculated using Supplementary equation (3).

Supplementary Note 4-Peripheries of the capillary bridges

For hydrophobic fibres, the capillary bridges formed at top of each fibre as shown in Supplementary Fig. 4a. A plane (red line) intersects the capillary bridges well above the fibre facet. The surface tension force acting on the plane is $\sum_{i=1}^{n}\oint_{s_i}\gamma\sin\theta_i ds_i$, where θ_i is localized contact angles for the ith fibre, s_i is the circumference of the ith quartz fibre, n is the total number of fibres. Due to the interaction between capillary bridges, the water contact angles varied along the periphery of the fibres within the range of $96.4° \le \theta < 180°$. The force generated by Laplace pressure acting on the plane is given by $\Delta P \times A_{\text{f}} \times n$, where A_{f} is the cross-sectional area of the fibre.

For hydrophilic fibres, a liquid column formed and wrapped the fibres as shown by a plane (red line) that intersects the capillary bridge well above the fibre facet in Supplementary Fig. 4b. We assumed the shapes of the base of the capillary bridges as rounded corner polygons shown in Supplementary Fig. 4c. The peripheries of these rounded corner polygons were used to estimate the adhesive forces of the hydrophilic-fibre MRS. When the fibre number is sufficiently low (equal to or less than six used in the present work, for example), all the fibres are at the periphery of the capillary bridge, thus the estimated adhesive force increases proportionally to the fibre number. When the fibre number is sufficiently large, however, some fibres would unavoidably be at the interior of the bridge so that the adhesive force would levels off as the fibre number increases.

During the thinning of the capillary bridge before the detachment of the water droplet, the angle θ between the capillary bridge and fibre facet decreased. When θ decreased to be $90°$, the surface tension force acting on the plane reached its maximum, $\gamma \times s$, where γ is the water surface tension, s is the circumference of the base of the capillary bridge. The force generated by Laplace pressure is given by $\Delta P \times A_{\text{c}}$, where ΔP is Laplace pressure, A_{c} is the cross-sectional area of the base of a capillary bridge.

Supplementary Note 5-The adhesion between droplets and the hydrophobic-fibre MRS

The hydrophobic-fibre MRS is composed of a superhydrophobic mesh and hydrophobic fibres. Water droplets have negligible adhesion on the superhydrophobic mesh, and are pinned by the fibres, due to the adhesion between the water droplets and the smooth hydrophobic fibres. Here, our hydrophobic fibres have a water receding contact angle θ of $96.4°$. The solid-liquid adhesion is given by the Young-Dupré equation as following[2]:

$$\gamma_{\mathrm{LG}}(1+\cos\theta)=\gamma_{\mathrm{SG}}+\gamma_{\mathrm{LG}}-\gamma_{\mathrm{SL}}>0\ \ (\theta<180°) \tag{4}$$

where indices L, G, and S of interfacial energies γ represent liquid, gas, and solid, respectively. Therefore, the adhesion between the hydrophobic microfibres and water is always larger than zero. Such adhesion is sufficient to pin tiny droplets.

To demonstrate the solid-liquid adhesive force between hydrophobic surfaces and water droplets, we made a pendant water droplet in contact with a hydrophobic substrate which has a water contact angle of 108° (Supplementary Fig. 5a). During the retreating of the microsyringe tip, the pinning force acted on the water droplet caused the pinch-off of the droplet. For a superhydrophobic substrate, a water droplet readily detached from the surface (Supplementary Fig. 5b).

Supplementary Note 6-Nearly-loss-free release of water droplets

For the 3-hydrophobic-fibre MRS, during the withdrawing of the microfibres, the water contact line easily receded on sides of the fibres. Once the contact line moved to the edge of the fibres, it was pinned initially. According to Gibbs criterion, the three phase contact line started to recede again when the receding angle was reached on the top facet during detaching. As a result, we observed the remaining water with volume of roughly 0.07 nl on the top of a hydrophobic fibre, as shown in Supplementary Fig. 6a.

For the 3-hydrophilic-fibre MRS, the superhydrophobic mesh repelled water and the receding contact angle was reached on sidewalls of the fibres during the withdrawing of the microfibres. The water contact line receded, leaving no trace of water behind. Right before the detachment of the water droplet from the 3-fibre MRS, the liquid column was split into three small capillary bridges on each fibre facet. After the pinch-off of the three capillary bridges, there was roughly 0.15 nl water residue on the top of each fibre, as shown in Supplementary Fig. 6b.

Supplementary Note 7-Assisting detection of highly-diluted chemicals

The detection of highly-diluted chemicals bears great interest in fields such as medical diagnostics, pollution detection and biomedicine synthesis. To detect traces of chemicals, the evaporation of sample droplets on a superhydrophobic detection substrate is proved to be an effective method; however, the requirement for the superhydrophobicity of the detection substrates impairs generality of the method because most of detection substrates such as lots of surface-enhanced Raman spectroscopy (SERS) substrates are difficult to be made highly water-repellent.

The capability of the MRS for capturing and releasing both micro- and nano-litre droplets with nearly no loss shows potential for assisting the detection of highly-diluted chemicals or biomarkers[3, 4]. As shown in Supplementary Fig. 7, we used the MRS to capture a 9 μl droplet, which contained a fluorescent dye with an undetectable concentration, 66.7 nM rhodamine 6G (R6G). The droplet was evaporated under room temperature for 115 min. The volume of the droplet drastically shrank 225 times to 40 nl; consequently the concentration of the dye increased to roughly 15 μM, which is clearly detectable under a fluorescence microscope. Such application of assisting the detection of low-concentration substances cannot be easily achieved by using a microsyringe or micropipette.

Supplementary Note 8-Mixing between droplets for micro-reactors

The mixing process of a coalesced droplet was recorded by a high speed camera (Supplementary Fig. 8a). Once merged, extra surface energy was released which converted into inertial energy causing the coalesced droplet oscillated violently[5]. The oscillation induced internal convection flow which improved the mixing process. After 5.5 seconds, the colour of the coalesced droplet became uniform.

Furthermore, the mixing can be easily enhanced by introducing a gentle air flow surrounding the pendant droplet (Supplementary Movie 4). For demonstration, a water droplet of 4 μl, containing titanium dioxide particles for visualizing the internal flow, was attached to the MRS. Then, nitrogen flow with a slow speed of around 10 cm s^{-1} blew towards the pendant droplet. Under such low gas flow, a rapid shear-driven convection immediately occurred inside the droplet, promoting the mixing.

The size distribution of silver nanoparticles synthesized in Fig. 6b was determined based on the TEM image (Supplementary Fig. 8b). The synthesized silver nanoparticles have an average diameter of 7.87 nm with a standard deviation (SD) of 3.54 nm (Supplementary Fig. 8c). The size distribution is in agreement with the UV-vis absorption spectrum which has a peak at 389 nm with full width at half maximum (FWHM) of 70 nm (Supplementary Fig. 8d)[6].

Supplementary References

1. Zhao, X. D., *et al.* Electrically adjustable, super adhesive force of a superhydrophobic aligned MnO2 nanotube membrane. *Adv. Funct. Mater.* **21**, 184-190 (2011).
2. Israelachvili, J. N. *Intermolecular and surface forces: revised third edition*. Academic press (2011).
3. De Angelis, F., *et al.* Breaking the diffusion limit with super-hydrophobic delivery of molecules to plasmonic nanofocusing SERS structures. *Nat. Photon.* **5**, 682-687 (2011).
4. Mclane, J., Wu, C., Khine, M. Enhanced detection of protein in urine by droplet evaporation on a superhydrophobic plastic. *Adv. Mater. Interf.* **2**, (2015).
5. Lai, Y.-H., Hsu, M.-H., Yang, J.-T. Enhanced mixing of droplets during coalescence on a surface with a wettability gradient. *Lab Chip* **10**, 3149-3156 (2010).
6. Mulfinger, L., Solomon, S. D., Bahadory, M., Jeyarajasingam, A. V., Rutkowsky, S. A., Boritz, C. Synthesis and study of silver nanoparticles. *J. Chem. Educ.* **84**, 322 (2007).
